# What determines cognitive estimation ability? Changing contributions of semantic and executive domains as a function of age

**DOI:** 10.1111/jnp.12279

**Published:** 2022-05-21

**Authors:** Paul Hoffman, Sarah E. MacPherson

**Affiliations:** ^1^ School of Philosophy, Psychology & Language Sciences University of Edinburgh Edinburgh UK

**Keywords:** cognitive ageing, cognitive estimation, executive function, semantic cognition

## Abstract

The Cognitive Estimation Test (CET) is commonly used in neuropsychological assessment. It is typically assumed to load on executive functions, although research has shown that CET performance also depends on access to semantic knowledge. It is unknown whether these contributions vary with age. It is important to examine this question as these abilities have divergent life course trajectories: executive functions tend to decline as people age but semantic knowledge continues to accrue. In addition, previous research has not examined potential contributions to CET performance from semantic control abilities, that is cognitive control processes involved specifically in the retrieval and use of semantic information. To address these questions, we investigated cognitive predictors of CET performance in healthy young and older adults. We found that better executive function was associated with more accurate estimation in both age groups. However, the effect of semantic knowledge on CET performance was significantly larger in older people, having no predictive power in the younger group. The ability to detect weak semantic associations, which is thought to index controlled search and retrieval of semantic information, also had divergent effects on CET performance in the two age groups. Our results provide empirical support for the idea that older people are more reliant on semantic knowledge when estimating quantities, which may explain why age‐related decline in CET scores is not typically found. We conclude that deficits on the CET may be indicative either of semantic or executive impairments, particularly in older age groups.

## INTRODUCTION

In everyday life, we are frequently required to generate reasonable estimates of quantities whose exact value is unknown to us (e.g. estimating the number of people who might attend a seminar we are planning). Producing estimates is a complex task that loads on various cognitive abilities, including the ability to use prior knowledge and experience in novel and flexible ways, to develop and apply strategies in estimation and to monitor the appropriateness of responses (MacPherson, Wagner, Murphy, Bozzali, & Cipolotti, 2014; Shallice & Evans, 1978). The Cognitive Estimation Task (CET) is commonly used to assess this ability. Various versions of the CET exist, all of which require participants to generate estimates for everyday quantities (e.g. what is the length of an average man's spine?) (Axelrod & Millis, 1994; Brand, Kalbe, Fujiwara, Huber, & Markowitsch, 2003; Bullard et al., 2004; Della Sala, MacPherson, Phillips, Sacco, & Spinnler, 2003; Scarpina, D'Aniello, Mauro, Castelnuovo, & MacPherson, 2015). Test scores reflect the degree to which estimates are reasonable (i.e. fall within the normal distribution of responses made by healthy controls) and/or the frequency of extreme under‐ or overestimates that are highly inappropriate. Deficits in CET performance have been reported in a range of conditions such as stroke (Shoqeirat, Mayes, MacDonald, Meudell, & Pickering, 1990), Alzheimer's disease (Della Sala, MacPherson, Phillips, Sacco, & Spinnler, 2004), frontotemporal dementia and corticobasal syndrome (Bisbing et al., 2015), Korsakoff's syndrome (Brand et al., 2003), Huntington's disease (Brandt, Folstein, & Folstein, 1988), traumatic brain injury (Schretlen, 1992) and psychiatric conditions such as schizophrenia (e.g. Gansler, Varvaris, Swenson, & Schretlen, 2014; Roth et al., 2012).

Clinically, the CET is typically used as a test of executive function (Strauss, Sherman, & Spreen, 2006). Executive functions refer to higher‐order control processes that allow individuals to formulate and control their behaviour to attain their desired goals, especially when facing novel or difficult situations (e.g. Stuss & Levine, 2002). To provide suitable estimates, individuals must recognise and choose the appropriate way of thinking or interpreting information, retrieve and manipulate precise details or estimates, check how suitable their response is and repeat this process if a better estimate is required. The frontal lobes are known to play a critical role in executive functions (e.g. Stuss & Alexander, 2000) and CET deficits are frequently observed following frontal lobe damage (Cipolotti et al., 2018; Della Sala et al., 2004; Shallice & Evans, 1978; Smith & Milner, 1984). However, other studies have not found a significant difference between frontal and nonfrontal patients performing the CET (Stanhope, Guinan, & Kopelman, 1998; Taylor & O'Carroll, 1995), suggesting that CET performance depends on more distributed brain networks. In addition, some studies have failed to find correlations between CET performance and scores on other executive measures (e.g. Appollonio et al., 2005; Barabassy, Beinhoff, & Riepe, 2010; D'Aniello, Scarpina, Albani, Castelnuovo, & Mauro, 2015; Spencer & Johnson‐Greene, 2009), leading some to argue that the CET does not specifically tap executive functions (D'Aniello et al., 2015),

Successful performance on the CET is also likely to depend upon semantic knowledge being intact and retrievable. For example, to provide an appropriate estimation for the item, ‘What is the maximum speed of a cheetah?’, one could access semantic knowledge about the speeds of various animals or modes of transport to compare against. Indeed, tests that probe forms of semantic knowledge, such as vocabulary, reading ability and general knowledge, are also correlated with CET performance (Della Sala et al., 2004; Gillespie, Evans, Gardener, & Bowen, 2002; MacPherson et al., 2014; O'Carroll, Egan, & MacKenzie, 1994). These results suggest that multiple cognitive domains contribute to cognitive estimation. While an individual's level of executive function is likely to be an important determinant of their CET performance, it seems that individuals with a rich store of semantic knowledge to draw on are also at an advantage when estimating real‐world quantities.

The present evidence suggests that both executive functions and semantic knowledge contribute to CET performance. However, it is not known whether the relative contribution of these two domains changes as people grow older. In fact, executive functions and semantic knowledge do have divergent trajectories with respect to age. According to neuropsychological models of cognitive ageing, the cognitive changes associated with healthy adult ageing are primarily due to deterioration in the frontal lobes (MacPherson & Cox, 2017; MacPherson, Phillips, & Della Sala, 2002; Moscovitch & Winocur, 1992; West, 1996). A considerable number of studies have reported age‐related performance declines in executive functions using both standard neuropsychological tests and experimental paradigms (Argiris, MacPherson, Della Sala, & Foley, 2020; Daigneault, Braun, & Whitaker, 1992; Lamar & Resnick, 2004; MacPherson et al., 2002; Mittenberg, Seidenberg, O'leary, & DiGiulio, 1989). Older age is associated with poorer performance on executive tests including the Wisconsin Card Sorting Test (WCST; Ashendorf & McCaffrey, 2008) the self‐ordered pointing task (Lamar & Resnick, 2004), the Tower tests (Allamanno, Della Sala, Laiacona, Pasetti, & Spinnler, 1987) and the Stroop task (Van der Elst, Van Boxtel, Van Breukelen, & Jolles, 2006). Yet, while neuropsychological models of cognitive ageing would predict that older adults should also perform more poorly than younger adults on the CET given it is an executive task, older adults have been found to perform as well as or better than younger adults on the CET (Axelrod & Millis, 1994; Della Sala et al., 2003; Gillespie et al., 2002; MacPherson et al., 2014).

Semantic knowledge, on the contrary, continues to accrue throughout the life course, with older people regularly achieving higher scores than young people on tests of vocabulary and general world knowledge (Hoffman, 2018; Park et al., 2002; Salthouse, 2004; Verhaeghen, 2003). Della Sala et al. (2004) suggested that, when making estimates, healthy older people may use their more developed semantic knowledge to compensate for poorer executive function. This factor could explain the preservation of CET performance in later life. If true, this would have implications for clinical assessment, as a CET deficit could indicate different forms of underlying cognitive impairment depending on the age of the person being assessed. It is also relevant to a wider question of central importance in cognitive ageing, namely: what are the relative contributions of executive ability vs. existing knowledge to complex cognitive tasks, and how do these change as people grow older?

Here, we investigated the degree to which executive and semantic abilities predict CET performance in healthy young vs. older adults. In addition to measuring participants' breadth of semantic knowledge and their general executive function, we included measures of semantic control ability. Semantic control refers to people's ability to exercise cognitive control in order to use their semantic knowledge in a flexible and goal‐oriented fashion (Jefferies, 2013). Semantic knowledge and semantic control make independent contributions to performance on semantic tasks, have distinct neural correlates and can dissociate under brain damage (Hoffman, Rogers, & Lambon Ralph, 2011; Jefferies & Lambon Ralph, 2006; Lambon Ralph, Jefferies, Patterson, & Rogers, 2017). Semantic control appears to rely partly on domain‐general executive processes and partly on more specialised mechanisms (Whitney, Kirk, & o'Sullivan, Lambon Ralph, & Jefferies, 2011); thus, it is important to assess this ability separately from domain‐general executive functions. There is also evidence that aspects of semantic control decline with age, even as semantic knowledge continues to accrue (Hoffman, 2019; Krieger‐Redwood et al., 2019; Wu & Hoffman, 2022). However, the specific contributions of semantic control abilities to the CET have not previously been assessed. In the present study, we assessed two forms of semantic control that are often contrasted with one another and have been linked with distinct neural correlates in the prefrontal cortex. These were the ability to retrieve weak semantic associations and the ability to select between competing semantic representations (Badre & Wagner, 2007). In so doing, we aimed to investigate which particular aspects of semantic control are important for CET performance, beyond the contribution of general executive functions.

## Method

### Participants

One hundred and six young adults, aged between 17 and 30 years, were recruited from the undergraduate Psychology course at our university and participated in the study in exchange for course credit. Data from six participants were excluded because they had not responded attentively during the semantic tasks, resulting in a final sample of 100 (see Results for details). Eighty‐six older adults, aged between 60 and 90 years, were recruited from the Psychology department's volunteer panel at our university and participated voluntarily. No older participants were excluded due to inattention. Participants were included if they reported being native speakers of English and had lived in the UK for the majority of their lives. Participants reporting a history of dyslexia, substance abuse or stroke were excluded. Participants were also asked to report whether they had ever suffered from a head injury or a neurological or psychological illness. 35% of young participants and 15% of older participants answered yes or maybe to these questions. We did not exclude these individuals because the wording of the questions did not allow us to discriminate current and historical conditions or between clinically diagnosed and self‐diagnosed conditions. To ensure that inclusion of these participants did not affect our results, we included medical history as a binary covariate in all mixed effects analyses.

Demographic information for each group is shown in Table 1. Older adults had completed significantly more years of education than participants in the young group. In addition, both groups were characterised by a preponderance of female participants relative to male, though this was more pronounced in the young group (*χ* ^2^ = 14.3, *p* < 0.001). Because of these group differences, education and sex were included as covariates in our analyses of CET performance. Informed consent was obtained from all participants, and the study was approved by our local Research Ethics Committee.

**TABLE 1:** Demographic characteristics and mean task performance by age group

	Young	Older	*t*
Age	18.5 (1.5)	69.5 (6.6)	‐‐
Years of education	13.3 (0.9)	15.2 (2.8)	6.58***
Sex (M:F)	10:90	29:57	‐‐‐
Cognitive estimation test/54	38.3 (5.7)	42.0 (5.1)	4.65***
Lexical decision (% correct)	77.2 (6.3)	92.9 (5.5)	17.90***
Lexical decision (RCS)	35.5 (8.5)	38.4 (11.2)	2.02*
Vocabulary scale (% correct)	37.0 (10.3)	71.4 (13.8)	19.40***
Vocabulary scale (RCS)	5.7 (2.1)	9.7 (4.1)	8.53***
Weak associations (% correct)	83.5 (7.9)	93.9 (5.1)	10.50***
Weak associations (RCS)	21.3 (5.0)	16.6 (4.6)	6.58***
High semantic selection (% correct)	81.5 (14.9)	76.0 (24.9)	1.85
High semantic selection (RCS)	16.7 (4.5)	10.7 (4.7)	8.83***
WCST (% correct)	82.8 (6.4)	76.5 (12.5)	4.45***
WCST (RCS)	37.7 (8.4)	22.7 (8.0)	12.50***

*Note:* Standard deviations are reported in parentheses. *p < 0.05; ***p < 0.001.

Abbreviations: RCS = rate correct score (correct responses per minute on task). WCST = Wisconsin Card Sorting Test.

### Cognitive estimation test

Participants completed the updated version of the CET devised by MacPherson et al. (2014). MacPherson et al. reported data for two parallel forms of the test. To maximise the available data for each participant, here, we combined both forms to make a single test with 18 questions. Each question required participants to estimate an unknown quantity (*e.g*. ‘How fast do race horses run?’). Participants were asked to provide an estimate in the measurement units of their choice, and then, answers were converted into the same unit of measurement for scoring purposes. They were instructed that this was a test of estimation, and they were not expected to know the exact answers. They were asked to give an estimate for each question, even if it was a guess. They were given an example question and estimate before beginning the test (What is the height of the Eiffel Tower? 150 m). No time limit was placed on responses, and participants checked a box to agree that they would not discuss their estimates with other people or research their answers on the internet.

Responses were scored for accuracy using the method and normative data provided by MacPherson et al. where each response was compared with the distribution of participants' responses in the normative sample. However, rather than calculating a CET error score, we calculated an accuracy score where the higher the score, the better the CET performance to ease comparisons with the other tasks used in the study. Therefore, a response that fell below the 5th percentile or above the 95th percentile was considered very extreme and received 0 points. A response within this range but outside the 10th/90th percentile was awarded 1 point. A response within this range but outside the 20th/80th percentile was awarded 2 points. Finally, responses falling between the 20th and 80th percentile were awarded the maximum 3 points. On a small number of trials (0.7%), participants failed to give a valid unit for their estimate or gave a unit that made no sense in the context of the question (e.g. ‘What is the maximum speed of a Harley‐Davidson motorbike?’ 2 m). These invalid trials were excluded from further analysis. For each participant, their mean accuracy score over all valid trials was multiplied by 18 to give them a total CET score out of a maximum of 54.

### Tests of semantic and executive ability

Participants also completed a semantic and executive test battery first reported by Hoffman (2018). The breadth of participants' semantic knowledge was assessed using two vocabulary tests. The first was the Spot‐the‐Word test (Baddeley, Emslie, & Smith, 1992), a two‐alternative lexical decision task. The second was an adapted version of the Mill Hill vocabulary scale (Raven, Raven, & Court, 1989), in which participants were presented with 44 low‐frequency words and asked to choose a synonym for each one from four alternatives.

Two semantic control tasks tested participants' ability to exercise cognitive control during semantic processing. In both tasks, participants were presented with a probe word and asked to choose a semantically related word from either two or four alternatives. Unlike the vocabulary tests, the stimuli were common words expected to be within the vocabulary of all participants (Hoffman, 2018). In the global association task, participants made judgements based on overall semantic relatedness. Need for control was manipulated by varying the strength of the association between the probe and its associate. In the strong association condition (24 trials), the correct item was closely associated with the probe (*e.g. house‐home*), whereas in the weak association condition (24 trials), the relationship was more distant (*e.g. house‐tent*). Weak associations are assumed to require more cognitive control in order to retrieve the relevant semantic information to identify the connection (Badre & Wagner, 2007). Trials were scored correct if the participant selected the semantically related item.

In the feature association task, participants were asked to match items based on specific attributes. At the beginning of each block of trials, participants were given a feature (e.g. colour) and were asked to match items based on that feature (*e.g. tomato‐blood*). Need for control was manipulated by varying the degree of competition between the correct response and distractors. In the low selection condition (24 trials), the correct response had a strong pre‐existing association with the probe (*e.g. cloud‐snow*), while the distractors were unrelated. In the high selection condition (24 trials), the probe and target shared no meaningful relationship other than their similarity on the specified feature (*e.g. salt‐dove* are both typically white but otherwise semantically unrelated) and, in addition, one of the distractors shared a strong semantic relationship with the probe (*salt‐pepper*). Thus, the high selection condition required greater control in order to focus on the relevant relationship between the probe and target and inhibit the strong but irrelevant distractor. In this task, trials were scored correct if the participant selected the item that matched on the relevant feature.

Finally, a computerised version of the Wisconsin Card Sorting Test (WCST) was used as a measure of non‐semantic executive ability. This was based on the test provided on the Psytoolkit website (https://www.psytoolkit.org/experiment‐library/wcst.html) and consisted of 64 trials. Trials were scored correct when participants correctly used the card‐sorting rule currently in operation.

### Procedure

Participants completed all tasks in a single online session, presented through a web browser using Psytoolkit (Stoet, 2017). Tests were completed in the following order: CET, semantic knowledge, semantic control, WCST. Response times were recorded for all of the tests except the CET, and participants were instructed to respond as quickly as possible without making mistakes.

### Statistical analyses

The primary aim of our analysis was to investigate which measures of semantic and executive ability predict CET performance and whether these predictors vary with age group. Predictors were semantic knowledge (average of the two vocabulary tasks), the two high‐control semantic conditions (weak semantic associations and high semantic selection) and the WSCT. For all of these abilities, we computed two performance measures for each participant. The first was simply mean accuracy over all trials. In order to take speed as well as accuracy into account, the second was a rate correct score (RCS) calculated by dividing the number of correct responses by the total time taken to complete all trials (Woltz & Was, 2006). This score represents the number of correct responses made per minute on the task. Reaction times falling more than two standard deviations outside a participant's mean were winsorised before computing the total task time.

Outlying scores on each cognitive ability were trimmed by winsorising values that fell more than two standard deviations outside each age group's mean. Scores were then entered into a series of mixed effects models which predicted CET accuracy on a trial‐by‐trial basis. Since CET accuracy was an ordinal response variable (each trial could receive a score of 0, 1, 2 or 3), cumulative link mixed models were fitted using the Ordinal package in R. All models included random intercepts for participants and items and fixed effects of education, sex and medical history. Models with a fixed effect of age group also included a random by‐items slope for the effect of age group. This allowed for the possibility that age had different effects on different questions in the CET. Continuous predictors were standardised by conversion to z‐scores prior to model entry.

We first fitted a model that predicted CET performance based on age group, along with the control predictors and random effects. We then proceeded to construct a model that predicted CET performance based on each of the four semantic/executive abilities and their interactions with age group. As we had two performance measures for each ability (accuracy and RCS) and these were strongly correlated with one another, for each ability, we selected the measure that provided the best fit to the data. To determine this, we estimated two mixed models for each ability, which were identical except that one used the accuracy score and the other the RCS score. Each model also included accuracy and RCS scores for the other three abilities. The model with the lowest AIC value was taken as the best fitting and the measure in this model was taken forward to be included in the final model. The final model therefore included a single measure for each semantic/executive task. We compared this final model with a maximal model that included both measures for each ability. The maximal model did not fit the data better than the single‐measure model (*χ* ^2^[8] = 2.31, *p* = 0.97), and it had a higher AIC score, suggesting it was less parsimonious.

Finally, we performed supplementary analyses of performance on the semantic control tasks, in order to assess the replicability of findings reported by Hoffman (2018). Hoffman (2018) investigated how different forms of semantic control were affected by ageing. He found that older people were more affected than young people by increased competition in the feature association task (high vs. low selection), suggesting that this aspect of semantic control declines with age. However, there were no effects of age on the manipulation of association strength (strong vs. weak), suggesting that controlled retrieval of weak semantic links does not decline with age. To test the replicability of these effects, we fitted a binomial generalised linear mixed effects model with accuracy as the dependent variable and age group, task (global vs. feature) and control demands (strong association/low selection vs. weak association/high selection) as fixed factors. The model also included a fixed effect of medical history, random effects of participant and items and random slopes for all factors varying within participants or items. The significance of effects was tested using a likelihood ratio test comparing the full model to a reduced model that excluded the effect being tested. Follow‐up models were estimated for global and feature tasks separately using the same approach.

## RESULTS

### Attention checks

Prior to the full analysis, we analysed the distribution of RTs to identify and exclude participants who did not appear to be completing the tasks attentively. We examined RTs on the Vocabulary Scale as this task was the most difficult. The mean RT over all trials was 4322 ms. RTs of less than 1 s were considered to be indicative of poor engagement with the task. RTs <1 s occurred on 2.7% of trials overall. Most participants made few if any such responses, but six participants produced responses <1 s on more than 20% of trials (all young). These participants were excluded on the basis that they did not appear to be engaged with the study.

### Effects of age on task performance and correlations between tasks

**FIGURE 1:**
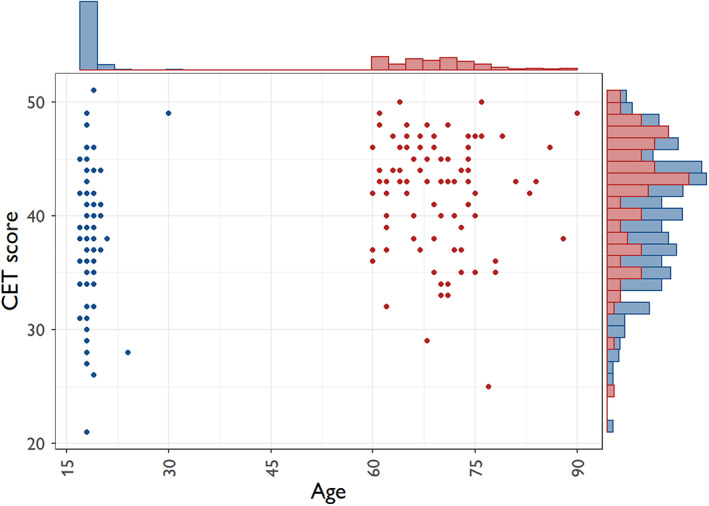
Distribution of ages of participants in the study and their CET scores. Blue = young group; red = older group

Figure 1 shows the distribution of CET scores and ages over all participants. Table 1 shows the mean scores for each age group on all tasks. Older people scored significantly higher on the CET than young adults. They also outperformed young people on the tests of semantic knowledge (lexical decision and vocabulary), regardless whether performance was scored in terms of accuracy or the RCS measure that also took speed into account (correct responses per minute). When judging weak semantic associations, older people were more accurate than young people but young participants exhibited higher RCS scores, suggesting greater efficiency in making these judgements at the expense of accuracy. The young group also performed better on the high semantic selection trials for the RCS measure, and this was accompanied by a non‐significant advantage in accuracy. Finally, for the WCST, the young group outperformed the older group on both accuracy and RCS. In summary, these results indicate an advantage for older people on the semantic knowledge tasks but a more mixed pattern of performance on the semantic control tasks. In addition, older people demonstrated poorer non‐semantic executive ability.

Correlations between measures within each age group are shown in Table 2. Given that the scores on the two semantic knowledge tasks were strongly correlated (*r* = 0.56 in young people and *r* = 0.81 in older people), these scores were averaged and the mean semantic knowledge score is considered from this point onwards. In the older group, CET score was positively correlated with semantic knowledge and WCST performance, as well as semantic selection. In contrast, in the young group, CET was only significantly positively correlated with the WSCT RCS measure.

**TABLE 2:** Correlations between measures in the young group (below diagonal) and older group (above diagonal)

	Age	Edu	CET	Know. %	Weak Assoc. %	Sem Select. %	WCST%	Know. RCS	Weak Assoc. RCS	Sem Select. RCS	WCST RCS
Age	‐	−0.31**	−0.05	0.11	−0.15	−0.27*	−0.20	−0.15	−0.44***	−0.43***	−0.41***
Education (yrs)	0.24*	‐	0.23*	0.54***	0.27*	0.33**	0.20	0.51***	0.32**	0.43***	0.35***
CET	0.15	−0.07	‐	0.35**	0.00	0.18	0.23*	0.27*	0.12	0.25*	0.33**
Knowledge %	0.09	0.13	−0.03	‐	0.39***	0.22*	0.28*	0.63***	0.16	0.23*	0.28*
Weak Assoc. %	0.25*	0.15	0.16	0.33***	‐	0.19	0.23*	0.28**	0.38***	0.21*	0.24*
Semantic selection %	0.04	−0.02	−0.03	0.25*	0.20*	‐	0.56***	0.16	0.14	0.75***	0.45***
WCST %	0.04	0.00	0.15	0.11	0.01	−0.06	‐	0.19	0.15	0.48***	0.77***
Knowledge RCS	−0.07	−0.04	0.08	0.25*	0.05	−0.08	0.16	‐	0.65***	0.52***	0.35***
Weak Assoc. RCS	−0.04	−0.11	0.15	0.05	0.31**	−0.09	0.16	0.61***	‐	0.63***	0.38***
Semantic selection RCS	−0.03	−0.18	0.03	0.18	0.06	0.52***	0.09	0.43***	0.55***	‐	0.55***
WCST RCS	0.07	−0.02	0.22*	−0.17	0.01	−0.16	0.52***	0.26**	0.40***	0.27**	‐

Abbreviations: Edu, education; CET, Cognitive Estimation Test; Know., knowledge; Weak Assoc., weak associations; Sem Select., semantic selection; WCST, Wisconsin Card Sorting Test; %, percent correct responses; RCS, rate correct score (correct responses per minute on task).

*p < 0.05; **p < 0.01; ***p < 0.001.

### Effects of semantic and executive abilities on CET performance

Performance on the CET was predicted using cumulative link mixed effects models that predicted accuracy scores (0–3) at the single‐trial level. The first model tested for an effect of age group, with sex, education and medical history included as covariates. As expected, there was a significant age effect (*z* = 2.25, *p* = 0.025) in favour of older people. Sex also had a significant effect on performance, with males scoring better than females (*z* = 2.73, *p* = 0.006). There were no effects of education (*z* = 1.19, *p* = 0.23) or medical history (*z* = 0.15, *p* = 0.88). We then proceeded to fit a model that included the four semantic/executive abilities and their interactions with age group. The model selection process indicated that accuracy scores were the better predictors for semantic knowledge and weak associations, while RCS values were optimal for the semantic selection and WCST tasks. Therefore these measures were used in the final model.

**FIGURE 2:**
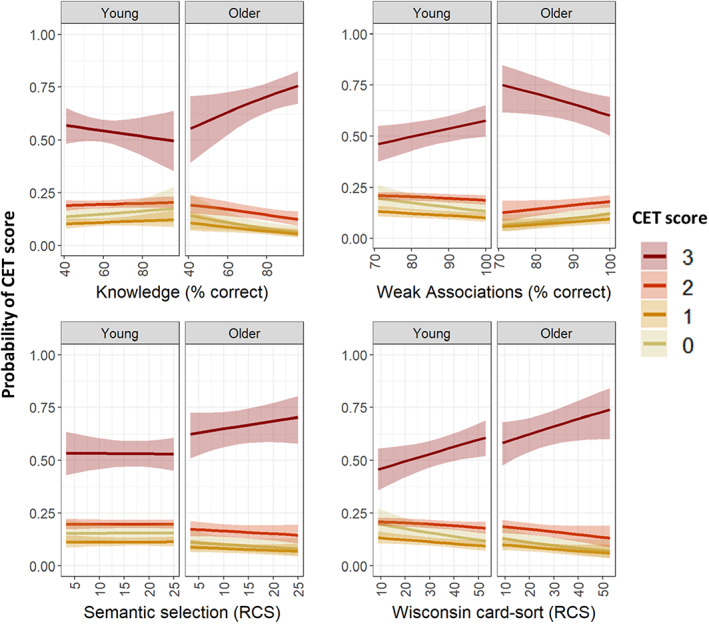
Predicted CET scores as a function of group and test scores. Figure shows predicted probability of achieving scores of 0–3 on each CET question. The higher the score, the better the CET performance. Shading indicates 95% confidence intervals. RCS, rate correct score (correct responses per minute on task)

Model coefficients are reported in Table 3, and predicted CET scores as a function of each predictor are shown in Figure 2. The only ability to have a main effect on CET performance was WCST. As seen in Figure 2, participants who performed better on the WCST were more likely to receive the highest accuracy scores when making estimates on the CET. Effects were similar in both age groups. In addition, however, there were significant interactions between age group and both semantic knowledge and weak association scores, indicating that these abilities had different effects in the two age groups. To investigate these effects further, follow‐up models were estimated for each age group separately. These omitted the effects of age group and their interactions but were otherwise identical to the main model. These models indicated that semantic knowledge had no effect on CET performance in young people (*z* = −0.36, *p* = 0.72) but had a marginal effect in older people (*z* = 1.86, *p* = 0.063). This effect was in the expected direction: older people with better semantic knowledge tended to make more accurate estimates. For the weak association task, in young people, better performance on this task predicted better CET scores (*z* = 2.04, *p* = 0.04). In contrast, in the older group, there was a tendency for people with higher weak association scores to perform worse on the CET (*z* = −1.90, *p* = 0.058)

**TABLE 3:** Mixed effects model predicting accuracy on CET trials

Effect	B	*SE*	*z*	*p*
Sex	−0.126	0.052	2.44	0.015*
Education	−0.033	0.054	0.62	0.538
Medical history	−0.020	0.040	0.51	0.611
Group	−0.273	0.101	2.71	0.007*
Semantic knowledge (%)	0.085	0.083	1.02	0.308
Weak associations (%)	−0.029	0.058	0.50	0.615
Semantic selection (RCS)	0.044	0.054	0.81	0.416
WCST (RCS)	0.163	0.061	2.67	0.008*
Group * semantic knowledge (%)	−0.160	0.082	1.97	0.049*
Group * weak associations (%)	0.157	0.058	2.73	0.006*
Group * semantic selection (RCS)	−0.044	0.055	0.81	0.420
Group * WCST (RCS)	−0.009	0.061	0.14	0.887

Abbreviations: RCS, rate correct score (correct responses per minute on task); WCST, Wisconsin Card Sorting Test.

### Semantic control manipulations

**FIGURE 3:**
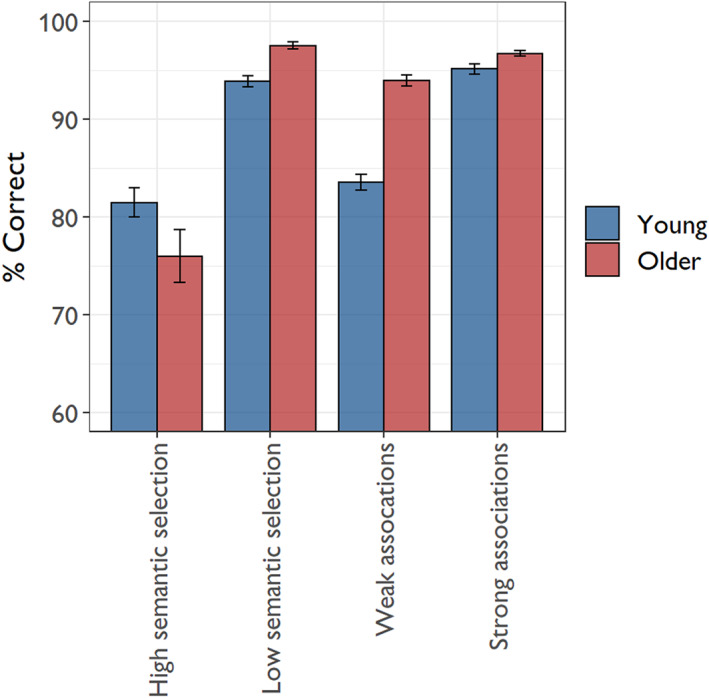
Accuracy on semantic control tasks as a function of age and control demands. Error bars represent one standard error of the mean

Finally, we performed a more detailed analysis of performance on the semantic control manipulation to test whether the present results replicate effects of age on semantic control, as previously reported by Hoffman (2018). Accuracy data were analysed with a 2 × 2 × 2 mixed model that included age group, control demands (strong/low selection vs. weak/high selection) and task (global vs. feature) as factors. The results are shown in Table 4, and the means for each condition are presented in Figure 3. As expected, participants were less accurate in the conditions requiring greater controlled processing. Older people also performed better overall, and performance was better for the global semantic judgements. Importantly, however, there was a significant three‐way interaction between the three factors, indicating that age group had divergent effects on the control manipulations in the two tasks. Separate analyses of each task indicated that, in the feature task, the increase in semantic selection demands had a greater detrimental effect on the older age group (control x group interaction: *χ* ^2^ = 9.87, *p* = 0.002). As shown in Figure 3, older people were less accurate than young people when making semantic judgements with high selection demands, despite outperforming them in the low selection condition. However, in the global task, the manipulation of associative strength did not interact with group (control x group interaction: *χ* ^2^ = 0.30, *p* = 0.58). As shown in Figure 3, older people were more accurate than young people when identifying both strong and weak associations (main effect of group: *χ* ^2^ = 22.1, *p* < 0.001). These results closely replicate those reported by Hoffman (2018). They indicate that older people have difficulty when required to exercise control to select among competing sources of semantic information. At the same time, older people show no deficits in the ability to retrieve weak semantic associations.

**TABLE 4:** Results of mixed effects model predicting accuracy on semantic control tasks

Effect	Chi‐square	*p*
Age group	22.54	<0.001
Task	13.36	<0.001
Task * age group	9.56	0.002
Control	44.23	<0.001
Control * age group	1.57	0.211
Task * control	4.83	0.028
Task * control * age group	6.58	0.010
Medical history	1.72	0.189

## Discussion

Estimating everyday quantities is a complex cognitive task that relies on the interaction of semantic knowledge with executive control processes. We investigated the contribution of semantic and executive abilities to performance on the CET, a commonly used neuropsychological test of estimation, and tested how these contributions vary as a function of age. Non‐verbal executive ability, indexed by the WCST, was a consistent predictor of CET performance in both young and older adults. However, the influence of semantic abilities varied across age groups. Breadth of semantic knowledge was a stronger predictor of CET scores in older people, having no predictive power in young people. The ability to detect weak semantic associations also had distinct effects in different age groups. Young people who were better at detecting weak associations between words performed better on the CET while the reverse appeared to be true for older people. These results confirm that cognitive estimation is supported by a combination of executive and semantic abilities but suggest that their relative contributions change across the lifespan.

A number of previous studies have reported that CET scores are correlated with performance on tests of semantic knowledge (Della Sala et al., 2004; Gillespie et al., 2002; MacPherson et al., 2014). Here, we used a battery of semantic tasks that distinguished between breadth of semantic knowledge and two aspects of controlled semantic processing. We found that the effect of semantic knowledge on the CET varied as a function of age, with only older participants showing a positive effect of this variable. This provides empirical support for the notion that older people rely to a greater extent on semantic knowledge when estimating quantities (Della Sala et al., 2004). This would explain why levels of semantic knowledge predicted how well older people performed on the CET, but they had no predictive influence in young people. It is possible that this greater reliance on semantic knowledge compensates for poorer executive function in older people; indeed, we did find significantly poorer WCST performance in our older group.

The ability to identify weak semantic associates also had divergent effects on CET scores in younger and older people. Detection of weak associates is thought to require controlled search through semantic memory in order to retrieve the relevant aspects of knowledge to identify the link between the items (Badre & Wagner, 2007; Hoffman, McClelland, & Lambon Ralph, 2018). This controlled retrieval function is primarily supported by regions of inferior prefrontal cortex (Badre, Poldrack, Pare‐Blagoev, Insler, & Wagner, 2005). We found that this ability had more a positive effect on CET performance in young people. This difference might reflect different strategies employed by different age groups, according to the knowledge available to them. Since older people have a more extensive set of semantic knowledge, they may in some cases have been able to retrieve the correct answer without having to estimate (e.g. they may have learned at some point the fact that racehorses typically run at around 40 miles per hour). In contrast, young people were less likely to have learned an exact answer and were more likely to need to construct a novel estimate ‘on the fly’. In these circumstances, we propose that young people need to search for any pieces of weakly related knowledge they have that could be used to form a reasonable estimate. Thus, for young people, high skills in controlled retrieval of knowledge may be more important in producing accurate estimates.

This account does not explain, however, why older people showed a trend towards *negative* effects of weak associates’ performance on the CET. Although we do not have a complete explanation for this finding, we speculate that it may be related to older people's poorer ability to inhibit irrelevant semantic information. In our semantic battery, we found that older people performed less accurately than young people on high semantic selection trials, replicating previous findings (Hoffman, 2018). It is therefore possible that older people do not benefit from retrieving weakly related semantic information because they find it difficult to select relevant facts from the retrieved information to use in generating estimates. However, this account predicts that people with poor selection ability would perform more poorly on the CET and we found no evidence for this. Thus, further research is needed to understand how semantic control processes influence estimation in older people, particularly as the observed effect of weak associates performance was only marginal in significance (*p* = 0.058).

Our current study replicates MacPherson et al. (2014) in that better CET performance is associated with older people and male participants. However, unlike MacPherson et al., we did not find independent effects of education on performance. This may be due to limited variance in years of education in our study, particularly in the younger group who were all university students and hence had similar backgrounds. Indeed, one of the advantages of MacPherson et al. (2014) was the inclusion of a large number of participants whose levels of education varied greatly. Importantly, we did find that executive function, as measured by the WCST, was predictive of CET scores in both age groups. This supports previous studies that have reported a relationship between estimation abilities measured by the CET and executive functions (Brand et al., 2003; Shoqeirat et al., 1990; Spencer & Johnson‐Greene, 2009), including the WCST (Liss, Fein, Bullard, & Robins, 2000). These findings advocate for the widespread use of the CET as a measure of executive function.

Our results have implications for clinical practice because they suggest that CET deficits in older patients could arise from either a deficit in executive functions or an impairment to semantic knowledge. Indeed, poor cognitive estimation has been reported in patients with semantic dementia, who suffer from a progressive and selective loss of semantic knowledge. Julien et al. (2010) found that semantic dementia patients were impaired in their estimation of real‐world quantities like those used in the CET, despite normal performance in more elementary estimations of physical quantities (see also Heim, McMillan, Olm, & Grossman, 2020). CET deficits are also correlated with tests of semantic memory in Alzheimer's disease, as well as executive function and working memory, underscoring the multi‐componential nature of the task in older populations (Brand et al., 2003; Levinoff et al., 2006).

The findings in our older group may also be driven by compensatory (cognitive reserve) abilities that go beyond any effects of age on semantic knowledge (Stern, 2012). Indeed, single‐word reading tests that are used as proxies of cognitive reserve may also be associated with semantic knowledge (Juncos‐Rabadán, Facal, Rodríguez, & Pereiro, 2010). Recent work has found that tasks that allow participants to use semantic and/or executive skills to improve performance show particularly strong effects of cognitive reserve (Darby, Brickhouse, Wolk, & Dickerson, 2017). However, the interaction between semantic knowledge and cognitive reserve remains unclear (Paolieri, Marful, Morales, & Bajo, 2018). While it is likely that both semantic knowledge and cognitive reserve contribute to CET performance, future work should examine the unique contribution of these factors.

It is important to note some further limitations of the present work that could be addressed in future studies. First, we only used one test of general executive function, the Wisconsin Card Sorting Test. While this task is commonly used as a measure of executive control, executive functions can be subdivided into different components (e.g. shifting, updating and inhibition; Miyake et al., 2000). The use of a wider range of tasks to measure different facets of executive control could provide further insights into the cognitive underpinnings of the CET and help to distinguish semantic control effects from more general executive functions. Our study was cross‐sectional, meaning that differences between age groups observed here may partly reflect cohort effects. Our two participant groups were both highly educated, and it is not clear the degree to which our conclusions would generalise to less‐educated populations. We found no main effect of education on CET performance in the present study, though previous studies have found a small positive relationship (Axelrod & Millis, 1994; Della Sala et al., 2003; MacPherson et al., 2014; O'Carroll et al., 1994). In addition, although we asked participants whether they had ever experienced neurological and psychological illness and included this factor as a covariate in our analyses, we were not able to distinguish between current and historical conditions. This meant we could not exclude participants currently experiencing mental ill health. Finally, we did not have access to brain scans for our participants. Future studies could use such information to investigate the neuroanatomical correlates of CET performance in healthy populations.

To conclude, the current study demonstrated that better executive function was associated with better CET performance regardless of participant age; however, there were differential effects of semantic abilities on CET performance in younger and older adults. Semantic knowledge significantly predicted CET performance in older people, but did not predict performance in the younger group. In terms of controlled semantic retrieval, there were also divergent effects of the ability to identify weak semantic associations on CET performance in the two age groups. These findings provide support for the notion that older people rely more on semantic knowledge when estimating quantities, and this may explain why age‐related decline on the CET is not found. We conclude that deficits on the CET may signify semantic or executive impairments, particularly in older adults.

## AUTHOR CONTRIBUTIONS

**Paul Hoffman:** Conceptualization; methodology; Investigation; Formal Analysis; Writing – original draft. **Sarah E. MacPherson**: Conceptualization; methodology; Writing – review & editing.

## CONFLICT OF INTEREST

There are no conflicts of interest.

## Data Availability

The data that support the findings of this study are available on request from the corresponding author. The data are not publicly available because participants did not consent to this.
